# The CAPN10 Gene Is Associated with Insulin Resistance Phenotypes in the Spanish Population

**DOI:** 10.1371/journal.pone.0002953

**Published:** 2008-08-13

**Authors:** María E. Sáez, José L. González-Sánchez, Reposo Ramírez-Lorca, María T. Martínez-Larrad, Carina Zabena, Alejandro González, Francisco J. Morón, Agustín Ruiz, Manuel Serrano-Ríos

**Affiliations:** 1 Neocodex, Departamento de Genómica Estructural, Seville, Spain; 2 Hospital Clínico San Carlos, Departamento de Medicina Interna II, Madrid, Spain; 3 Centro Avanzado de Fertilidad (CAF), Unidad de Reproducción y Genética Humana, Cádiz, Spain; Mayo Clinic College of Medicine, United States of America

## Abstract

Cardiovascular disease is the leading cause of morbidity and mortality in the industrialized world. Familial aggregation of cardiovascular risk factors is a frequent finding, but genetic factors affecting its presentation are still poorly understood. The calpain 10 gene (*CAPN10*) has been associated with type 2 diabetes (T2DM), a complex metabolic disorder with increased risk of cardiovascular disease. Moreover, the *CAPN10* gene has been associated with the presence of metabolic syndrome (MS) in T2DM and in polycystic ovary syndrome (PCOS). In this work, we have analysed whether the polymorphisms UCSNP44, -43, -19 and -63 are related to several cardiovascular risk factors in the context of MS. Molecular analysis of *CAPN10* gene was performed in 899 individuals randomly chosen from a cross-sectional population-based epidemiological survey. We have found that *CAPN10* gene in our population is mainly associated with two indicators of the presence of insulin resistance: glucose levels two hours after a 75-g oral glucose tolerance test (OGTT) and HOMA values, although cholesterol levels and blood pressure values are also influenced by *CAPN10* variants. In addition, the 1221/1121 haplogenotype is under-represented in individuals that fulfil the International Diabetes Federation (IDF) diagnostic criteria for MS. Our results suggest that *CAPN10* gene is associated with insulin resistance phenotypes in the Spanish population.

## Introduction

The *CAPN10* gene is located at 2q37 and encodes a ubiquitously expressed member of the calpain cysteine protease family. Horikawa *et al*. [1] positionally cloned *CAPN10* as type 2 diabetes mellitus (T2DM) gene in Mexican American and Finnish populations. In that study, the common G allele of UCSNP43 polymorphism and the haplotype combination 112/121 (that comprises UCSNP43, -19 and -63 polymorphisms) were associated with an increased risk of T2DM. Since then, the role of *CAPN10* has been examined in different ethnic groups with uneven results [2]. Genetic heterogeneity between populations is thought to be responsible for the different polymorphisms and haplotype combinations at *CAPN10 locus* that have been found associated with an increased risk of T2DM. In fact, a report by Fullerton *et al*. [3] has shown an unusual degree of geographic structure at this locus, especially at UCSNP19 and UCSNP63 sites, suggesting positive natural selection acting either on *CAPN10* gene or on closely linked *locus.* Despite these differences, two meta-analyses have confirmed the association of the UCSNP43 G allele with a higher risk of T2DM, whereas showed that the effect of the 112/121 haplogenotype was over-estimated in the initial studies [4], [5]

Genetic studies have associated UCSNP43, UCSNP19 and the haplotype combination reported by Horikawa *et al.* [1] with insulin levels [6]–[9]. Some of these genetic associations are accompanied by physiological studies that support the CAPN10 role in proinsulin processing, insulin secretion and insulin action [10]–[14]. In this way, other insulin related disorders have been linked to *CAPN10* gene such as polycystic ovary syndrome (PCOS). PCOS is a common endocrine disorder of women of reproductive age, characterized by chronic anovulation, infertility and hyperandrogenemia. This syndrome, in which insulin resistance is considered a key feature, has been independently associated with *CAPN10* allelic variants by Erhman *et al.* [15] and our group [16], [17] and has been recently confirmed in a meta-analysis [18].

Metabolic syndrome (MS) is characterized by abdominal obesity, atherogenic dyslipidemia, raised blood pressure, insulin resistance/glucose intolerance, prothrombotic and proinflammatory state [19]. Currently insulin resistance is considered the underlying mechanism for these metabolic alterations [20], [21]. The *CAPN10* gene has been associated with several components of MS such as hypertension in Chinese [22], [23] and African-American [24], elevated body mass index (BMI) in Japanese [25], cholesterol levels in Chinese [26] and hipertrigliceridemia in obese Swedish individuals [27]. Recently, the 111/121 haplotype combination has been associated with an increased risk of metabolic syndrome in Korean T2DM patients [28]. However, an analysis of this gene in individuals that fulfil the diagnostic criteria of MS in general population has not been performed.

The aim of this study was to analyse the role of *CAPN10* allelic variants in genetic susceptibility to MS and related metabolic traits in a Spanish population-based survey. Our main outcome is the identification of association between *CAPN10* gene variants and two estimators of insulin resistance: the glucose levels after an oral glucose tolerance test (OGTT) and the HOMA index. In addition, we have observed association with blood pressure and cholesterol levels and with MS under the IDF definition. Altogether, our results contribute to the notion of *CAPN10* as an insulin resistance gene.

## Materials and Methods

### Patients

This population based study includes 899 non related Caucasian men (n = 427, 47.5%) and women (n = 472, 52.5%) who were recruited by a simple random sampling approach from a cross-sectional population-based epidemiological survey in the province of Segovia in Central Spain (Castille), aimed at investigating the prevalence of anthropometric and physiological parameters related to obesity and other components of MS [29]. The age of this population ranged between 35 and 76 years (mean age 54.03±11.69 years). Subjects with previous diagnosis of type 1 diabetes were excluded from the study. All subjects gave their written consent to participate in the study. The study protocol was approved by the Ethics Committee of the Hospital Clínico San Carlos of Madrid.

### Phenotype measurements

Anthropometric measurements included body mass index (BMI, kg/m^2^) and waist circumference (WC, cm). Systolic (SBP) and diastolic blood pressures (DBP) were measured three times in the seated position after 10 minutes of rest to the nearest even digit by use of a random-zero sphygmomanometer.

After an overnight fast period, 20 ml of blood were obtained from an antecubital vein without compression. Plasma glucose was determined in duplicate by a glucose-oxidase method adapted to an Autoanalyzer (Hitachi 704, Boehringer Mannheim, Germany). Total cholesterol, triglycerides (TG) and high-density lipoprotein cholesterol (HDL-c) were determined by enzymatic methods using commercial kits (Boehringer Mannheim, Germany). Low-density lipoprotein cholesterol (LDL-c) was calculated by the Friedewald formula. Serum insulin concentration was determined by RIA (Human Insulin Specific RIA kit, Linco Research Inc., St Louis MO, USA).

An oral glucose tolerance test (OGTT) using 75 g of glucose was performed according to the WHO recommendations and the results were interpreted in accordance with Genuth *et al.* [30]. Insulin resistance was estimated by the homeostasis model assessment (HOMA-IR) method according to the formula: Insulin (μU/ml)×Glucose (mmol/l)/22.5 [31], [32].

The metabolic syndrome status was established according to ATPIII definition (SM_ATPIII) [32]: presence of at least three components between abdominal obesity (WC≥102 cm men, 88 cm women), hipertrigliceridemia (≥150 mg/dl), hypertension (≥85/130 mmHg), HDL-c (<40 mg/dl men, <50 mg/dl women) and fasting glucose≥100 mg/dl. We have also classified subjects according to the recently International Diabetes Federation (IDF) worldwide definition of the metabolic syndrome for Europid populations (SM_IDF) [33]. In this last definition, central adiposity (defined as WC≥94 cm for men and ≥80 cm for women) is a prerequisite risk factor for the diagnosis of the syndrome; two of the following factors are also necessary: raised TG level (>150 mg/dl) or specific treatment for this abnormality; reduced HDL-c (<40 mg/dl males, <50 mg/dl females) or specific treatment; raised blood pressure (SBP≥130 mmHg or DBP≥85 mmHg) or treatment of previously diagnosed hypertension, raised fasting plasma glucose (FPG≥100 mg/dl) or previously diagnosed T2DM.

Table 1 summarize the basal characteristics of the study population, expressed as phenotypic means±standard deviations for the quantitative traits and crude prevalences for the dichotomic traits.

**Table 1:** Baseline characteristics of study subjects.

*CONTINUOUS TRAITS*	*Men (n = 427)*		*Women (n = 472)*	
	*Mean*	*SD*	*Mean*	*SD*
***Age (years)***	53.49	11.73	54.51	11.64
***BMI (kg/m^2^)***	27.56	3.33	27.63	4.70
***Waist circumference (cm)***	95.36	8.96	85.56	11.20
***SBP (mmHg)***	126.65	16.58	124.75	19.46
***DBP(mmHg)***	78.79	9.12	77.78	9.99
***Fasting glucose (mmol/L)***	5.26	1.68	4.89	1.28
***2h-glucose (mmol/L)***	5.97	2.31	6.19	2.23
***Fasting insulin (pmol/L)***	78.24	48.54	79.68	59.46
***HOMA***	3.07	2.10	3.07	2.97
***TGs (mmol/L)***	1.31	0.82	1.00	0.54
***Cholesterol (mmol/L)***	5.61	1.04	5.52	1.05
***HDL-c (mmol/L)***	1.39	0.41	1.63	0.47
***LDL-c (mmol/L)***	3.62	0.94	3.44	0.93
***DICHOTOMIC TRAITS***	**N**	**%**	**N**	**%**
***Smoking***	142	33.3	73	15.5
***Physical activity***	237	55.5	312	66.2
***Alcohol consumption***	347	81.3	184	39
***T2DM***	37	8.7	36	7.9
***SM_ATPIII***	69	17	108	23.9
***SM_IDF***	90	21.8	121	26.8

### DNA extraction

DNA extraction from 300 μl of frozen peripheral blood was performed in a MagNapure LC Instrument (Roche Diagnostics), using MagNa Pure LC DNA Isolation kit (Roche Diagnostics) according to the manufacturer's instructions. Aliquots of DNA at 5 ng/ul were obtained to carry out PCR reactions.

### Genotyping

We have determined allele status at the single nucleotide polymorphisms previously associated with metabolic syndrome traits UCSNPs-44,-43,-19 and -63 according the nomenclature and definitions reported by Horikawa *et al.* [1] and Evans *et al.* [34].

#### UCSNP44 and –43

These two SNPs were amplified in a same amplicon of 204 bp using previously described primers 43F/43R [16]. The reverse primer was biotin labeled for genotypes determination in a PSQ™ 96 (Pyrosequencing AB, Uppsala, Sweden). The internal sequencing primer was 5′ CAG GGC GCT CAC GCT TGC 3′. This instrument allows the simultaneous analysis of 96 samples using pyrosequencing techniques [35].

#### UCSNP19

This polymorphism was amplified using previously described forward primer (19F) [16] and new designed reverse primer (19R2: 5′-GCA GGG TCT AAG CAG CAG CTA C-3′). Amplicon sizes were 152 bp for allele 1 (2 repeats of 32 bp sequence) and 184 bp for allele 2 (3 repeats of 32 bp sequence) and were differentiated in 2% agarose gel.

#### UCSNP63

Allele status was determined using previously described procedure [16].

### Statistical Analysis

To analyze differences in genotype distribution, chi-square association studies for MS and T2DM and test for deviation of Hardy-Weinberg equilibrium (HWE), we have used tests adapted from Sasieni [36] at the online resource available at the Institute for Human Genetics, Munich, Germany (http://ihg.gsf.de).

For genotype quantitative analysis, we have performed an analysis of variance using the GLM procedure included in SPSS software (Ver. 11.0.0., LEAD Technologies, Inc). Normality of the dependent variables was assessed by Kolmogorov-Smirnov test and, when necessary, we applied mathematical transformations (natural logarithm or square root) prior to the analysis. The adjusted percentage of the phenotypic variance explained by each genotype was estimated by subtracting the adjusted R^2^ value for a model that includes the genotype from the R^2^ for a model that excludes it. The Levenés test of equality of error variances was also performed for each analysis. In addition, all values from individuals under antihypertensive, antidiabetic or hypocholesterolemic medication were eliminated of the QTL analysis.

Haplotype analysis was performed using the THESIAS software (http://www.genecanvas.org) based on the SEM algorithm [37]. This method allows to estimate haplotype frequencies and haplotype effects by comparison to a reference (the intercept) taken here as the most frequent one. Haplotypes effects are expressed as mean effects (increases/decreases of the phenotypic mean with respect to the intercept's one).

All the studies were adjusted for sex, age, BMI, smoking (defined as present or past history of smoking of at least five cigarettes per day for a minimum of 5 years), alcohol consumption (defined as a daily intake of more than 10 g), and physical activity. Interaction effects of age, sex and BMI were explored.

## Results

### Genotypic association analysis

Allele 1 frequencies at the four polymorphic *loci* within *CAPN10* gene (UCSNP44, -43, -19 and -63) in our population were 0.85 at UCSNP44 (allele T), 0.74 at UCSNP43 (allele G), 0.36 at UCSNP19 (2 repeats of 32 bp) and 0.95 at UCSNP63 (allele C) (Table 2). These allelic frequencies do not differ from previously determined by our group in healthy controls from Granada, Southern Spain (p>0.06) [16]. All genotypic frequencies fit those expected by the HWE (p>0.06), except for the UCSNP63 polymorphism, for which we detected a high degree of homozygosity (p = 0.002); genotypes of five out the eight individuals homozygous for the polymorphic allele at this locus were confirmed by direct sequencing.

**Table 2:** Genotype distribution at *CAPN10* UCSNP44, -43, -19 and -63 sites and analysis of Hardy-Weinberg equilibrium (HWE).

*SNP*	*F _ALlELE 1_*	*GENOTYPIC DISTRIBUTION*			*P _HWE_*
		11	12	22	
***UCSNP44 T>C***	0.85	637 (71.9%)	226 (25.5%)	23 (2.6%)	0.604
***UCSNP43 G>A***	0.74	495 (56.6%)	311 (35.6%)	68 (7.8%)	0.062
***UCSNP19 del/ins***	0.36	120 (13.7%)	396 (45.3%)	359 (41.0%)	0.511
***UCSNP63 C>T***	0.95	795 (90.3%)	77 (8.8%)	8 (0.9%)	0.002

Allele nomenclature according to Horikawa *et al.* [1] and Evans *et al.* [34]

The genotype distributions at UCSNP44 and UCSNP19 loci were shown to influence glucose levels after an OGTT (χ^2^_2 d.f._, p = 0.008 and p = 0.011 respectively), explaining each of them around a 3% of the phenotypic variance observed for this trait (Table 3). Individuals homozygous for the less frequent alleles C at UCSNP44 and 2-repeats allele at UCSNP19 loci were associated with a significant lower glucose levels when compared with the remaining genotypes; conversely, homozygotes for the more frequent alleles (T at UCSNP44 and 3-repeats allele at UCSNP19), have higher OGTT values. Both UCSNP44 and UCSNP19 polymorphisms are also associated with the HOMA index, an indirect measure of insulin resistance; this trait is also influenced by the UCSNP43 locus. The effect size observed in terms of HOMA variance explained by *CAPN10* polymorphisms is higher than the observed for the 2-hours glucose levels, being all of them over a 4.6%.

**Table 3:** Genotypic association analysis.

*PHENOTYPE*	*SNP*	*GENOTYPE*	*MEAN*	*SE*	*adjusted R2*	*P VALUE*
**2h-GLUCOSE ** ***(mmol/L)***	**UCSNP44**	**2 DF**			3.3	**0.008**
		**11**	5.89	<0.01	3.1	**0.012**
		**12**	5.58	<0.01	-	Ns
		**22**	4.75	0.01	3.0	**0.016**
	**UCSNP19**	**2 DF**			3.1	**0.011**
		**11**	5.39	<0.01	2.8	**0.014**
		**12**	5.75	<0.01	-	Ns
		**22**	6.01	<0.01	2.8	**0.014**
**HOMA index**	**UCSNP44**	**2 DF**			4.9	0.052
		**11**	2.77	0.03	5	**0.017**
		**12**	2.42	0.04	5	**0.017**
		**22**	2.58	0.12	-	Ns
	**UCSNP43**	**2 DF**			4.6	0.055
		**11**	2.62	0.03	-	Ns
		**12**	2.64	0.04	-	Ns
		**22**	3.22	0.07	4.7	**0.016**
	**UCSNP19**	**2 DF**			4.7	**0.048**
		**11**	2.41	0.05	-	Ns
		**12**	2.59	0.03	-	Ns
		**22**	2.84	0.03	4.7	**0.022**
**SBP (mmHg)**	**UCSNP19**	**2 DF**			2.4	**0.021**
		**11**	124.14	1.49	-	Ns
		**12**	120.44	0.86	2.5	**0.007**
		**22**	123.01	0.86	-	Ns
**DBP (mmHg)**	**UCSNP19**	**2 DF**			1.1	**0.015**
		**11**	78.30	0.89	-	Ns
		**12**	75.98	0.51	1.1	**0.005**
		**22**	77.56	0.51	-	Ns
**CHOLESTEROL ** ***(mmol/L)L***	**UCSNP63**	**2 DF**			0.5	0.098
		**11**	5.51	0.05	0.6	**0.016**
		**12**	5.80	0.13	0.5	**0.025**
		**22**	5.71	0.43	-	Ns
**LDL-cholesterol ** ***(mmol/L)***	**UCSNP19**	**2 DF**			1.9	0.060
		**11**	3.47	0.01	-	Ns
		**12**	3.62	0.06	2.1	**0.018**
		**22**	3.44	0.06	-	Ns
	**UCSNP63**	**2 DF**			1.4	0.079
		**11**	3.51	0.04	1.5	**0.027**
		**12**	3.78	0.12	1.5	**0.024**
		**22**	3.58	0.38	-	Ns

Only the association studies which obtained a p value under 0.05 are shown. SE: standard error; Ns: Not significant. All values are adjusted by age, sex, alcohol consumption, smoking, physical activity and BMI.

The UCSNP19 polymorphism was also associated with two other MS related phenotypes: blood pressure (BP) and LDL-c levels, although the contribution to the phenotype is more modest that the observed for OGTT and HOMA (1.1≥r^2^≤2.4). Heterozygotes for this variant have lower BP values despite increased LDL-cholesterol levels whereas the homozygous presence of allele 2 is associated with lower LDL-c levels.

We have also observed that homozygotes for the UCSNP63 wild type allele have lower total and LDL cholesterol levels (p≤0.027) but given the departure of the HWE observed at this locus, these results must be interpreted with caution.

We have not found any association with BMI, waist circumference, triglycerides or HDL-c values (data not shown). We have neither observed association with T2DM nor with MS_ATPIII, but the heterozygous presence of the UCSNP19 polymorphism was associated with a higher risk for MS_IDF (OR = 1.89 [1.04–3.44], p = 0.036). Interaction analyses of *CAPN10* variants with age, sex and BMI were not significant, suggesting that the effects of these polymorphisms do not differ substantially between subjects for these reasons.

### Haplotypic association analysis

The haplotype reconstruction showed five possible haplotypes (1121, 1221, 2111, 1111 and 1112 for UCSNP44, UCSNP43, UCSNP19 and UCSNP63) according to previous reports from other populations (Table 4). Haplotypic frequencies are 0.37 for 1121, 0.25 for 1221, 0.15 for 2111, 0.16 for 1111 and 0.06 for 1112. For performing the association analysis, *CAPN10* haplotypes were compared with a reference haplotype, taken here as the most frequent one (haplotype 1121). In this analysis, we only observed a significant contribution of the 2111 haplotype to the OGTT (p = 0.002), having the carriers of this haplotype 9.8% lower 2-hours glucose levels in accordance to the genotypic association analysis, since allele 2 at UCSNP44 locus only appears in this background.

**Table 4:** Haplotypic frequencies and association analysis with OGTT values in Spanish population

*HAPLOTYPE*	*F*	*N*	*MEAN (95%CI)*	*PHENOTYPIC EFFECT (95%CI).*	*P VALUE*
1121	0,37	270	3,10 (2,94 −3,27)	1 (Reference)	-
1221	0,25	183	3,14 (2,95 −3,33)	−0,04 (−0,23–0,30)	0.762
2111	0,15	110	2,80 (2,53 −3,07)	−0,30 (−0,63– −0,02)	**0.002**
1111	0,16	117	2,95 (2,68 −3,22)	−0,15 (−0,50–0,19)	0.289
1112	0,06	43	2,70 (2,24 −3,17)	−0,40 (−0,91–0,11)	0.145

F: population frequency.

### Haplogenotypic association analysis

Finally, we have assigned haplotype pairs to all study subjects and performed haplogenotypic association analysis. In our population we identified fifteen haplogenotypes ranging in frequency from 0.93% to 17.11% (Table 5). In order to estimate the global haplogenotypic effect in the association analysis, only the haplotype pairs with a population frequency over 0.05 were computed. The only trait for which this global test was significant was LDL-c levels (χ^2^_7 d.f._, p = 0.027), with two associated protective haplogenotypes (1221/1121 and 2111/1111), whereas a trend was observed for total cholesterol (χ^2^_7 d.f._, p = 0.054) and SBP (χ^2^_7 d.f._, p = 0.063). Given that LDL-c is the main fraction of total cholesterol, a high concordance is observed in the association analysis since 1221/1121 and 2111/1111 are also total cholesterol lowering haplogenotypes, although the effect size is lower.

**Table 5:** Haplogenotypic association analysis.

Haplogenotype	N	%	Glc120			HOMA			INSULIN			SBP			CHOLESTEROL			LDL-cHOLESTEROL		
			MEAN	SE	P value	MEAN	SE	P value	MEAN	SE	P value	MEAN	SE	P value	MEAN	SE	P value	MEAN	SE	P value
**1221/1121**	147	17.11	5.96	<0.01	-	2.80	<0.01	-	71.07	0.05	-	123.93	1.40	-	3.20	0.04	**0.021 (−4.2)**	5.36	0.09	**0.005 (−6.9)**
1121/1121	137	15.95	6.06	<0.01	-	2.78	<0.01	-	68.71	0.05	-	124.79	1.59	-	3.23	0.04	-	5.52	0.09	-
1121/1111	104	12.11	6.15	<0.01	-	2.76	<0.01	-	67.20	0.05	-	118.80	1.51	-	3.07	0.04	-	5.64	0.11	-
**2111/1121**	96	11.18	5.59	<0.01	-	2.38	<0.01	**0.037 (−15.3)**	62.03	0.07	**0.027 (−12.9)**	120.90	1.85	-	3.13	0.05	-	5.56	0.12	-
1221/1221	84	9.78	6.00	<0.01	-	3.08	0.01	-	71.47	0.07	-	122.63	1.97	-	3.17	0.05	-	5.76	0.13	-
**2111/1221**	68	7.92	6.02	<0.01	-	2.63	0.01	-	67.46	0.07	-	119.86	2.04	**0.024 (−3.8)**	3.10	0.05	-	5.68	0.14	-
1221/1111	55	6.40	5.86	<0.01	-	2.78	0.01	-	73.89	0.12	-	121.19	2.66	-	3.13	0.07	-	5.32	0.19	-
**2111/1111**	45	5.24	5.77	<0.01	-	2.39	0.01	-	65.21	0.10	-	126.33	3.05	**0.050 (+3.9)**	3.27	0.08	**0.033 (−6.4)**	5.20	0.15	**0.040 (−8.6)**
1121/1112	32	3.73	5.87	0.01	-	2.65	0.01	-	68.26	0.22	-	122.38	2.72	-	3.16	0.07	-	5.54	0.13	-
**2111/2111**	23	2.68	4.98	0.01	**0.016 (−18.4)**	2.82	0.02	-	70.72	0.45	-	123.03	4.41	-	3.18	0.11	-	5.54	0.20	-
1221/1112	21	2.44	5.33	0.01	-	2.21	0.01	-	64.61	0.33	-	118.35	3.75	-	3.06	0.10	-	5.87	0.25	-
1111/1111	17	1.98	5.63	0.01	-	2.36	0.02	-	63.19	0.36	-	125.82	5.12	-	3.25	0.13	-	5.67	0.34	-
1112/1111	12	1.40	4.75	0.01	-	2.37	0.03	-	49.31	0.23	-	123.70	4.78	-	3.20	0.12	-	5.81	0.37	-
2111/1112	10	1.16	5.05	0.02	-	2.41	0.04	-	67.39	0.89	-	118.55	4.23	-	3.07	0.11	-	5.91	0.30	-
1112/1112	8	0.93	6.08	0.01	-	2.24	0.03	-	63.67	0.70	-	122.21	5.89	-	3.16	0.15	-	5.74	0.46	-

Results are given as p-values. Numbers in brackets represents the percentages of increase or decrease of the phenotypic mean with respect to the remaining haplotypes. All values are adjusted by age, sex, alcohol consumption, smoking, physical activity and BMI.

For SBP only the 2111/1221 haplogenotype individual analysis was significant (p = 0.024), showing a 3.8% decrease with respect to the remaining haplogenotypes. The 2111/1121 haplogenotype was associated with a reduction in both insulin and HOMA values (12.9%, p = 0.027 and 15.3%, p = 0.037 respectively) whereas the homozygous presence of the 2111 is associated with a significant decrease (18.4%) of the glucose levels after an OGTT (p = 0.016).

Finally, the homozygous presence of the UCSNP19 polymorphism in the 1221/1121 haplogenotype is associated with a lower risk of suffering from MS_IDF (OR = 0.51 [0.26–0.99], p = 0.044). No association was observed with either MS_IDF or T2DM.

## Discussion

Complex disorders represent the majority of human diseases, but their genetic bases are still unclear. *CAPN10* gene has been associated with type 2 diabetes in some studies, but other analyses failed to reproduce this finding. It may be due to differences in effect size in different ethnic backgrounds, disease allele frequencies, marker allele frequencies and extent of linkage disequilibrium at *CAPN10 locus* between populations that also accounts for the diverse CAPN10 alleles associated with insulin resistance phenotypes in those studies. However, distinct meta-analyses and also functional studies have confirmed the role of *CAPN10* in insulin resistance phenotypes [4], [5], [12], [13], [38], [39].

We have observed increased levels of both 2-hours glucose and HOMA values associated with the UCSNP44 TT genotype. Conversely, homozygous presence of the allele C (allele 2) is associated with higher glucose tolerance. This allele seems to be the most determining one in our population, since the unique haplotype in which the allele 2 exists was the only associated in the haplotypic analysis. Moreover, all but one of the haplogenotypes associated to the different phenotypes analysed comprise this allele. In all cases, the allele C confers protection from cardiovascular risk, not only regarding OGTT, HOMA or even insulin levels, but also regarding BP and plasma cholesterol. However, given the low frequency of this allele, we can not draw final conclusions. Previous studies have described an association of the allele C at UCSNP44 locus with T2DM [14], [34], [38]. This divergence could be related to specific population genetic background or to random chance in our results due to low population frequency. Functional studies suggest that UCSNP44 is located in an enhancer element and might affect *CAPN10* expression [1]. Also this polymorphism is in perfect linkage disequilibrium (*r* ^2^ = 1) with a missense mutation Thr504Ala (SNP-110) and two polymorphisms in the 5′-UTR region (UCSNP134 and UCSNP135) [38].

The other outstanding polymorphism in our population is UCSNP19, which is also associated with the OGTT and HOMA values, increased in the homozygotes for the 3-repeats allele (allele 2), and with LDL-c levels, that are in contrast decreased in these individuals. This antagonistic action is also observed in the heterozygous state, resulting in lower BP values despite increased LDL-c levels. The UCSNP19 polymorphism has been previously associated with other insulin resistance syndrome, PCOS, and with cholesterol levels in these women [16]–[18].

We have not found evidence of association either of the UCSNP43 G allele or the 112/121 haplogenotype originally described by Horikawa et al [1] as T2DM susceptibility variants, with any of the metabolic related traits analysed in this work. By contrast, the UCSNP43 A allele (allele 2) which is in complete LD with UCSNP19 allele 2, seems to be related to higher HOMA values in our population, but only in the homozygous state. The unique haplotype in which the UCSNP43 allele 2 is present (haplotype 1221), when combined with the 1121 haplotype, is associated with a lower risk of MS_IDF suggesting that the effect of the UCSNP43 only exists in the homozygous state or that the association of the 1221/1121 with MS_IDF is due to the heterozygous presence of the UCSNP19 allele 2. However, the haplogenotypic analysis is the least powerful one due to reduced sample size in each analysed group, so we can not rule out the possibility of type 1 error, especially given that we have not applied correction for multiple testing.

We have observed increased total and LDL cholesterol values associated with heterozygosity at UCSNP63 locus, but given the high deviation from HWE observed in our population, we must interpret this result with caution. Interestingly, the T2DM and MS subpopulations are in HWE (p = 1.000 and p = 0.115, respectively) (Table 6). We have examined HWE at UCSNP63 *locus* in the report by Kang et al. [28]. We found that deviation from HWE (HWD) exists in this T2DM population, with an excess of allele 2 homozygotes (p = 0.0004) and an inbreeding coefficient F of 0.18. Conversely, in a recent analysis of *CAPN10* performed on 312 healthy Korean subjects, HWD is not observed (p = 0.543) [40]. In Caucasians, a *CAPN10* gene analysis in the Botnia region of Finland, shows that HWE is also altered in the control population (Finland) (p = 0.045) but is preserved in the T2DM population (p = 0.416) [9]. In a report by Lynn *et al.* [8], there is no evidence of HWD in a non diabetic British population (p = 0.170), but an independent analysis according to T2DM familial antecedents, shows that it is only true in those individuals with T2DM relatives, showing the individuals without T2DM familial antecedents again an excess of UCSNP63 allele 2 (p = 0.037). These data could reflect the existence of selective pressures and high geographic structure affecting *CAPN10 locus* identified by several reports in accordance with the thrifty genotype hypothesis [3], [41], [42]. This hypothesis posits that T2DM genes have been submitted to the effects of natural selection [43].

**Table 6:** Analysis of Hardy-Weinberg equilibrium (HWE) at UCSNP63 locus in different populations.

*POPULATION*	*STUDY*	*AFFECTION STATUS*	*GENOTYPE DISTRIBUTION Observed (Expected)*			*HWE p value (1 df)*
			*11*	*12*	*22*	
***Spanish***	This report	**Healthy**	550 (546)	55 (63)	6 (2)	**0.006**
		**T2DM**	69 (69)	4 (4)	0 (0)	1.0
		**MS_IDF**	189 (188)	19 (23)	2 (1)	0.115
		**All population**	795 (790)	77 (88)	8 (3)	**0.002**
***Korean***	Kang *et al.* 2006	**T2DM**	200 (185)	132 (161)	50 (35)	**0.0004**
***Korean***	Xu *et al.* 2006	**Healthy**	179 (177)	112 (115)	21 (19)	0.5433
***Finnish (Botnia)***	Orho-Melander *et al.* 2002	**Healthy**	258 (255)	34 (39)	4 (1)	**0.0469**
		**T2DM**	313 (311)	70 (73)	6 (4)	0.4133
		**Both**	571 (567)	104 (113)	10 (6)	0.0578
***British***	Lynn *et al.* 2002	**Healthy with FA**	115 (116)	29 (28)	1 (2)	1.0000
		**Healthy without FA**	106 (103)	28 (34)	6 (3)	**0.0371**
		**Both**	221 (218)	57 (62)	7 (4)	0.1703

FA: familial antecedents of T2DM.

In summary, our results support a role for *CAPN10* in MS and related metabolic traits. However, the physiological mechanisms underlying the genetic associations described in this and other reports are not entirely clear. The pleiotropic effects of CAPN10 could be related with the key role of insulin in the harmonization of glucidic and lipidic metabolisms. The identification of other *loci* interacting *CAPN10* could provide clues about the physiological pathways involved. In this way, genetic interaction between *CAPN10* and *CYP19* gene has been suggested to increases susceptibility to T2DM [44]. *CYP19* aromatase encodes an enzyme involved in the steroid hormones synthesis from cholesterol, a biological process in which insulin is known to take part [45]. Aromatase knockout mice (ArKO) show increased intra-abdominal fat distribution and reduction of glucose oxidation, suggesting an important role for this metabolic route in the maintenance of lipid homeostasis in both males and females [46]. Members of the calpain family are involved in several biological processes through the proteolitic regulation of numerous transcription factors, but the physiological substrates for CAPN10 are still unknown. Further analysis considering gene-gene interactions will help resolve these questions.
